# Roles of ES Cell-Derived Gliogenic Neural Stem/Progenitor Cells in Functional Recovery after Spinal Cord Injury

**DOI:** 10.1371/journal.pone.0007706

**Published:** 2009-11-06

**Authors:** Gentaro Kumagai, Yohei Okada, Junichi Yamane, Narihito Nagoshi, Kazuya Kitamura, Masahiko Mukaino, Osahiko Tsuji, Kanehiro Fujiyoshi, Hiroyuki Katoh, Seiji Okada, Shinsuke Shibata, Yumi Matsuzaki, Satoshi Toh, Yoshiaki Toyama, Masaya Nakamura, Hideyuki Okano

**Affiliations:** 1 Department of Physiology, Keio University School of Medicine, Tokyo, Japan; 2 Department of Orthopedic Surgery, Keio University School of Medicine, Tokyo, Japan; 3 Department of Orthopedic Surgery, Hirosaki University Graduate School of Medicine, Hirosaki, Japan; 4 Department of Neurology, Graduate School of Medicine, Nagoya University, Nagoya, Japan; 5 Kanrinmaru Project, Keio University School of Medicine, Tokyo, Japan; 6 Department of Rehabilitation Medicine, Keio University School of Medicine, Tokyo, Japan; 7 Department of Research Super Star Program Stem Cell Unit, Graduate School of Medical Science, Kyusyu University, Fukuoka, Japan; Chiba University Center for Forensic Mental Health, Japan

## Abstract

Transplantation of neural stem/progenitor cells (NS/PCs) following the sub-acute phase of spinal cord injury (SCI) has been shown to promote functional recovery in rodent models. However, the types of cells most effective for treating SCI have not been clarified. Taking advantage of our recently established neurosphere-based culture system of ES cell-derived NS/PCs, in which primary neurospheres (PNS) and passaged secondary neurospheres (SNS) exhibit neurogenic and gliogenic potentials, respectively, here we examined the distinct effects of transplanting neurogenic and gliogenic NS/PCs on the functional recovery of a mouse model of SCI. ES cell-derived PNS and SNS transplanted 9 days after contusive injury at the Th10 level exhibited neurogenic and gliogenic differentiation tendencies, respectively, similar to those seen *in vitro*. Interestingly, transplantation of the gliogenic SNS, but not the neurogenic PNS, promoted axonal growth, remyelination, and angiogenesis, and resulted in significant locomotor functional recovery after SCI. These findings suggest that gliogenic NS/PCs are effective for promoting the recovery from SCI, and provide essential insight into the mechanisms through which cellular transplantation leads to functional improvement after SCI.

## Introduction

Because the adult central nervous system (CNS) has limited potential for regeneration, spinal cord injury (SCI) results in severe dysfunction, such as paraplegia and tetraplegia. With the aim of regenerating the injured spinal cord, various intraspinal cellular transplants have been investigated, especially in the sub-acute phase after injury. This period, between the acute and chronic phases, is marked by the minimal expression of cytokines, and is likely to be amenable to transplantation therapy [1], [2], [3], [4], [5]. Embryonic stem (ES) cells, with their indefinite replication potential, pluripotency, and genetic flexibility, have attracted great interest, and methods for inducing their neural differentiation have been extensively studied [6]. ES cell-derived neural progenitors are currently one of the most promising cell sources for cell transplantation therapy for treating SCI. Although previous studies demonstrated that the transplantation of mouse ES cell-derived embryoid bodies [7] or human ES cell-derived oligodendrocyte progenitor cells [8] promotes overall functional recovery after SCI, the types of neural progenitor cells most effective for treating sub-acute phase SCI has been uncertain.

We recently reported that a low concentration of retinoic acid (10^−8^ M: low-RA) can efficiently induce caudalized neural progenitors in embryoid bodies (EBs) [9], and we established a neurosphere-based culture system of ES cell-derived neural stem/progenitor cells (NS/PCs) from low-RA-treated EBs, with midbrain to hindbrain identities [10]. These ES cell-derived primary neurospheres (PNS) mainly exhibit neurogenic differentiation potentials, whereas passaged secondary neurospheres (SNS) are more gliogenic, corresponding to changes in CNS development, in which neurogenic NS/PCs predominate early in gestation and gliogenic NS/PCs predominate in mid-to-late gestation. Here, taking advantage of this difference between neurogenic PNS and gliogenic SNS, we transplanted PNS and SNS into the injured spinal cord, examined the differentiation and growth properties of the grafted cells, and compared their effects on angiogenesis, axonal regeneration, and functional recovery after SCI. We also examined the survival and growth of the transplanted ES cell-derived NS/PCs using *in vivo*, live, bioluminescent imaging (BLI) to evaluate the tumorigenicity and safety of the grafted cells.

## Results

### Establishment of a Stable ES Cell Line Expressing CBR*luc* Luminescence and Venus Fluorescence

**Figure 1:**
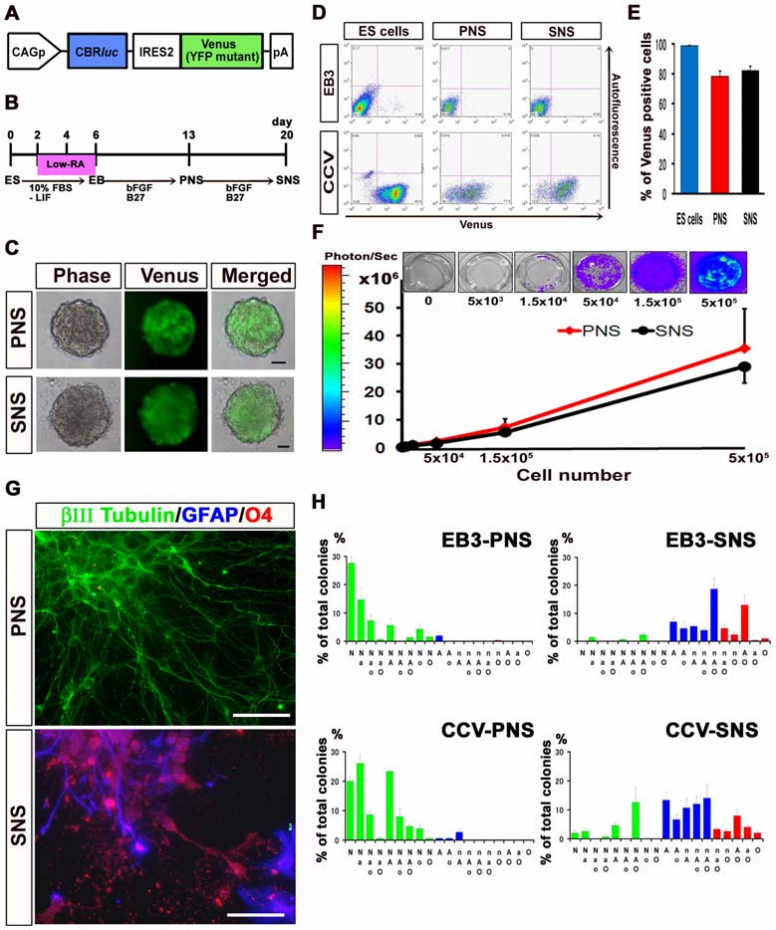
Establishment of a stable ES cell line expressing CBR*luc* luminescence and Venus fluorescence, and their differentiation analysis. (A) The CAG-CBR*luc*–IRES-Venus gene (CCV) construct. (B) Protocols for deriving neurospheres from mouse ES cells. ES cells were dissociated into single cells with 0.25% trypsin-EDTA and cultured for 6 days to allow the formation of embryoid bodies (EBs). A low concentration of RA was added on day 2 of EB formation for neural induction. The EBs were dissociated into single cells with 0.25% trypsin-EDTA and cultured in suspension for 7 days, to obtain primary neurospheres (ES cell-derived primary neurospheres, PNS). These PNS were dissociated into single cells with TripleLE Select (Invitrogen) and cultured again in suspension for 7 days under the same conditions to form secondary neurospheres (SNS). (C) Images of CCV-PNS and -SNS visualized by fluorescence microscopy. Scale bar: 100 µm. (D) Flow cytometric analysis of Venus-positive cells in PNS and SNS derived from CCV- and EB3-ES cells. (E) The proportion of Venus-positive cells among CCV-ES cells and their progenies, CCV-PNS and -SNS. Approximately 80% of the CCV-PNS and -SNS cells were positive for Venus. Values are means ± s.e.m. (*n* = 3). (F) Correlation between the cell numbers of CCV-PNS and -SNS, and the measured photon counts. BLI revealed CBR*luc* expression in both CCV-PNS and -SNS, and we determined that the photon counts were in direct proportion to the cell numbers *in vitro*. Values are means ± s.e.m. (*n* = 3). (G)(H) Distinct differentiation potentials of PNS and SNS *in vitro*. Immunocytochemical analysis of βIII tubulin-positive neurons (N or n), GFAP-positive astrocytes (A or a), and O4-positive oligodendrocytes (O or o) (N, A, O: more than 20 cells; n, a, o: fewer than 19 cells in each colony, respectively). A neuron-only colony (N) and a colony consisting of astrocytes and oligodendrocytes (AO) are shown (G). Scale bar: 50 µm. Quantitative analysis of the *in vitro* differentiation potential of EB3-PNS and -SNS and CCV-PNS and -SNS, shown as the percentage of each phenotypic colony among the total colonies (H). The PNS colonies dominantly differentiated into neurons, while a small number of colonies contained glial cells. On the other hand, most of the SNS colonies differentiated into both neurons and glial cells, including astrocytes and oligodendrocytes, or into only glial cells. Values are means ± s.e.m. (*n* = 3).

We first established an ES cell line that constitutively expresses the click beetle red-emitting luciferase (CBR*luc*) [11] and Venus [12] by introducing a CAG-CBR*luc*-IRES-Venus plasmid (Fig. 1**A**) into EB3 ES cells (CCV-ES cells) [13]. CCV-ES cells and their progenies were detected by both BLI [3], [14], [15] and fluorescence microscopy. To induce NS/PCs from ES cells and obtain PNS and SNS, we used a neurosphere-based culture system that we recently reported [10] (Fig. 1**B**), as described in Materials and Methods. More than 99% of the undifferentiated CCV-ES cells expressed Venus fluorescence by flow cytometry (Fig. 1**D and E**), and CCV-ES cell-derived PNS (CCV-PNS) and SNS (CCV-SNS) showed steady fluorescence that was detectable by fluorescence microscopy (Fig. 1**C**). Approximately 80% of the cells in the CCV-PNS and -SNS were positive for Venus by flow cytometry (Fig. 1**D and E**). bioluminescence imaging (BLI) revealed CBR*luc* expression in both CCV-PNS and –SNS, and we confirmed that the photon counts were in direct proportion to the cell numbers *in vitro* (Fig. 1**F**). We also confirmed that the CCV-ES cells could generate PNS and SNS similar to EB3-ES cells (Fig. 1**C**).

### Distinct Differentiation Potentials of PNS and SNS *In Vitro*

We next examined the *in vitro* differentiation potentials of the PNS and SNS derived from EB3- and CCV-ES cells. PNS and SNS derived from EB3- and CCV-ES cells were allowed to differentiate in medium without FGF2 on poly-L-ornithine/fibronectin coated coverslips for 5 days, and then processed for immunocytochemistry. We examined the frequency of colonies consisting of βIII tubulin-positive neurons, GFAP-positive astrocytes, and/or O4-positive oligodendrocytes, and found that the EB3- and CCV-PNS colonies predominantly differentiated into neurons, although a small number of colonies contained both neurons and glia (Fig. 1**G**). In contrast, most of the EB3- and CCV-SNS colonies differentiated into both neurons and glia, including astrocytes and oligodendrocytes, or into only glial cells (Fig. 1**G**), demonstrating that the ES cell-derived PNS and SNS had distinct differentiation potentials *in vitro* (Fig. 1**H**). Moreover, EB3- and CCV-ES cell-derived neurospheres exhibited similar differentiation properties, confirming that the transgene in the ES cells had negligible effects on differentiation (Fig. 1**H**).

We also examined the SNS formation rates to determine the self-renewing ability of the ES cell-derived PNS. We cultured CCV-PNS at a low cell density (2.5×10^4^ cells/ml), transferred them into 96-well plates at one neurosphere/well, dissociated the neurospheres, and cultured them again with FGF2 to form secondary neurospheres. Most of the CCV-PNS generated secondary neurospheres (79/90; 87.7%; from more than three independent experiments), confirming their ability to self-renew.

### Transplanted SNS Prevented Atrophic Change and Demyelination after SCI

**Figure 2:**
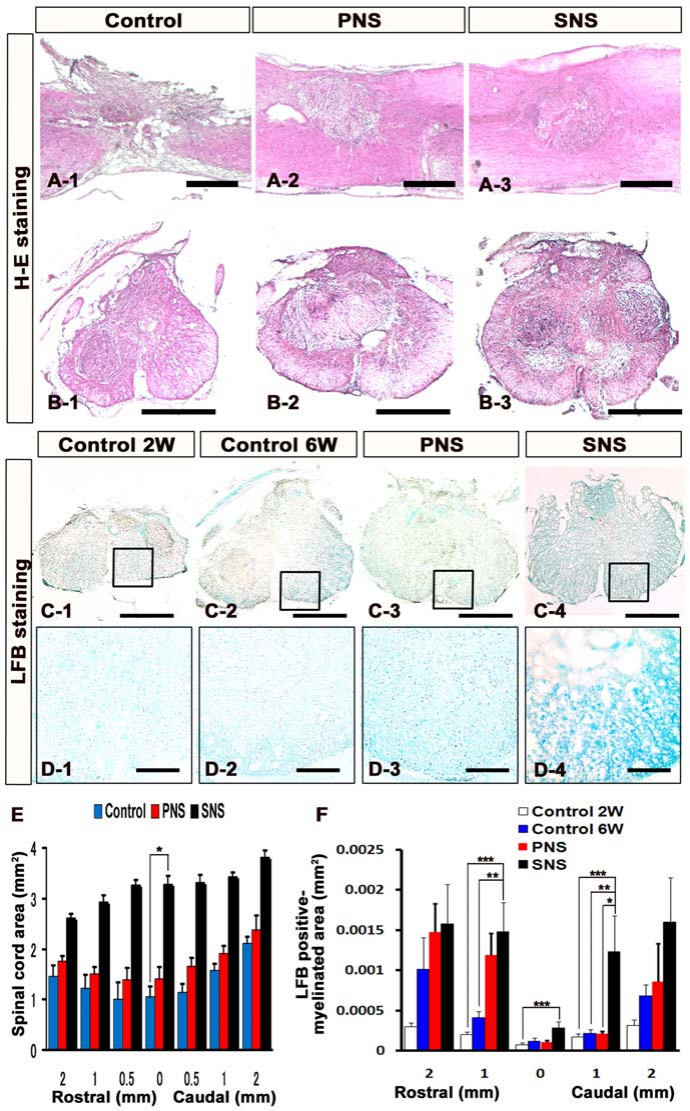
Transplanted SNS prevented atrophic change and demyelination after SCI. (A)(B) Representative H–E staining images of the sagittally sectioned (A1–3) and axially sectioned (B1–3) spinal cord at the lesion epicenter 6 weeks after injury. Scale bar: 500 µm. (C) Representative LFB staining images of the axially sectioned spinal cord 1 mm caudal to the lesion from each animal (2 weeks or 6 weeks after SCI for the vehicle-control group and 6 weeks after SCI for the PNS and SNS groups). Scale bar: 500 µm. (D) Higher magnification images of the boxed areas in C. Scale bar: 100 µm. (E) Quantitative analysis of the spinal cord area measured in H–E-stained axial sections through different regions. The transverse area of the spinal cord at the lesion epicenter was significantly larger in the SNS group compared with the control group. Values are means ± s.e.m. (*n* = 5). *: *P*<0.05, Control vs. SNS. (F) Quantitative analysis of the myelinated area by LFB-stained axial sections at different regions. LFB staining revealed greater preservation of myelination in the SNS group, with significant differences observed at the sites 1 mm rostral and 1 mm caudal to the epicenter compared with the control 2 or 6 weeks groups, 1 mm caudal to the epicenter compared with the PNS group, and at the epicenter compared with the control 2-week group. Values are means ± s.e.m. (*n* = 5). *: *P*<0.05, PNS vs. SNS. **: *P*<0.05, Control 6 weeks vs. SNS. ***: *P*<0.05, Control 2 weeks vs. SNS.

A contusive SCI was induced at the Th10 level of C57BL6 mice, and 5×10^5^ cells of CCV-PNS or CCV-SNS, or PBS as a control, were injected into the lesion epicenter 9 days after injury. We refer to these, respectively, as the PNS, SNS, and control groups. After 6 weeks, histological analyses were performed. We first examined atrophic changes of the injured spinal cord by Hematoxylin-eosin (H–E) staining (Fig. 2**A and B**). The transverse area of the spinal cord at the lesion epicenter was significantly larger in the SNS group than in the control group, suggesting that SNS transplantation prevented atrophy of the injured spinal cord (Fig. 2**E**). Luxol Fast Blue (LFB) staining revealed significantly greater preservation of the myelinated areas in the SNS group compared with the control (both 2 and 6 weeks after injury) and PNS groups (Fig. 2**C and D**), from 1 mm rostral to 1 mm caudal to the epicenter (Fig. 2F). Notably, there was a significantly spared rim of white matter in the SNS group, even at the lesion epicenter, whereas the control group exhibited severely demyelinated white matter throughout the lesioned area (2 mm rostral and caudal to the lesion epicenter) (Fig. 2**C and D**).

### Transplanted PNS and SNS survived in the injured spinal cord and did not form tumors

**Figure 3:**
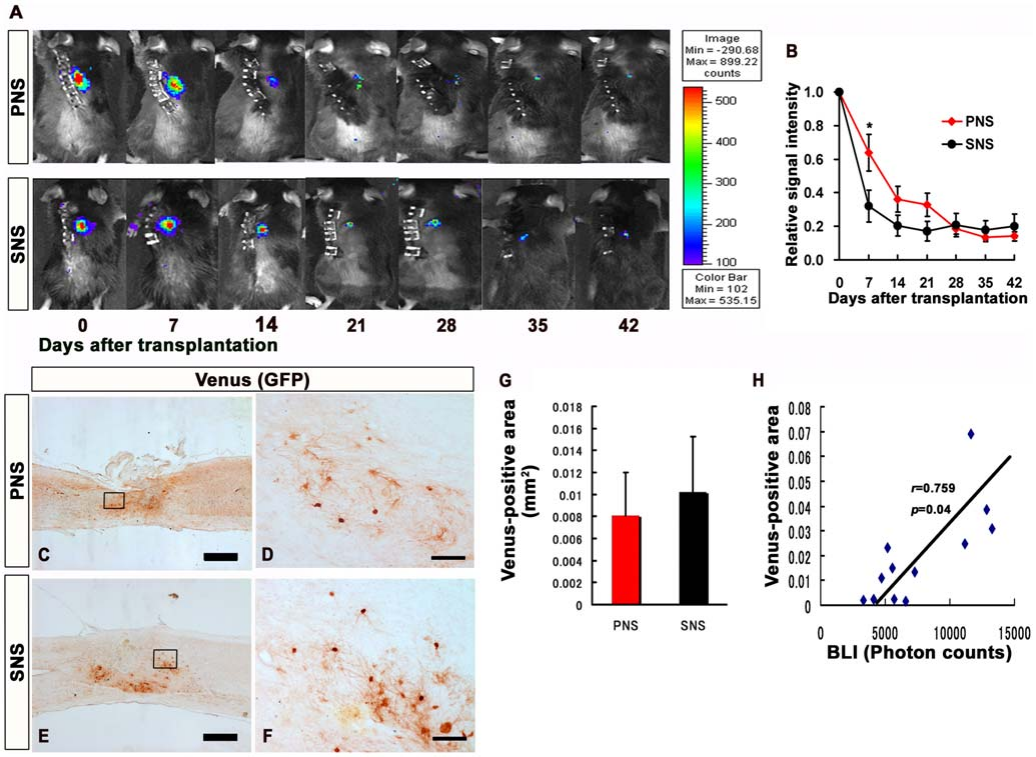
Transplanted PNS and SNS survived in the injured spinal cord and did not form tumors. (A) Representative BLI images of mice that received transplants of CCV-PNS and -SNS. (B) Signal intensity over time after transplantation in the PNS and SNS groups, shown relative to the initial value. Although the signal intensity at 1 week after the injury was significantly higher in the PNS group (62.4%) than the SNS group (29.5%), there was no significant difference in the signal intensity between the PNS (12.6%) and SNS (18.9%) groups 6 weeks after the injury. Values are means ± s.e.m. (*n* = 11). *: *P*<0.05, PNS vs. SNS. Scale bar: 500 µm. (C)(D)(E)(F) Representative images of sagittal sections of spinal cords grafted with PNS and SNS, which were immunostained for Venus (grafted cells) using an anti-GFP antibody. High-magnification images of the boxed areas in C and E are shown in D and F. Scale bar: 500 µm for C and E, 100 µm for D and F. Histological analysis confirmed that both PNS- and SNS-derived cells survived without forming tumors. (G) Quantitative analysis of Venus (GFP)-positive area at the lesion epicenter in midsagittal sections. Venus immunostaining revealed there was no significant difference between PNS and SNS groups 6 weeks after transplantation Values are means ± s.e.m. (n = 6). (H) Correlation of the results of BLI analysis and the quantification of Venus-positive area. The data of BLI correlated well with Venus-positive area (n = 12).

The photon count measured by bioluminescence imaging (BLI) quantifies only living cells, since the luciferin-CBR-luciferase reaction depends on oxygen and ATP. The successful transplantation of CCV-PNS and -SNS was confirmed immediately after transplantation using BLI, and the average signal intensity was 2.2±1.6×10^5^ photons/mouse/sec in 22 transplanted mice. Images obtained weekly thereafter for 6 weeks showed that the signal intensity dropped sharply within the first week after transplantation, but remained at 20% of the initial photon count in both the PNS and SNS transplantation groups throughout the remaining period. Although the signal intensity at 1 week was significantly higher in the PNS group (62.4%) than in the SNS group (29.5%), there was no significant difference in the signal intensity between the PNS (12.6%) and SNS (18.9%) groups at 6 weeks, suggesting there was a similar number of live grafted PNS- and SNS-derived cells within the injured spinal cord 6 weeks after transplantation. Thus, similar numbers of grafted PNS and SNS cells may have survived in the injured spinal cord, although the possibility that the grafted cells proliferated differently in the two groups 1 to 6 weeks after transplantation cannot be excluded. Notably, a rapid increase in signal intensity, which would have suggested tumor formation, was not observed during this time period (Fig. 3**A and B**). Consistently, histological analysis confirmed that both the CCV-PNS- and CCV-SNS-derived Venus-positive cells survived without forming tumors (Fig. 3**C**–**F**). Quantitative analysis of the Venus-positive area revealed that there was no significant difference of the number of survived grafted cells between PNS and SNS groups 6 weeks after transplantation (Fig. 3**G**). Moreover, the data of BLI correlated well with Venus-positive area (Pearson's correlation coefficient: 0.759, *p* = 0.04, Fig. 3**H**).

### PNS and SNS Grafted onto Injured Spinal Cord Exhibited Differentiation Potentials Similar to Those Observed *In Vitro*

**Figure 4:**
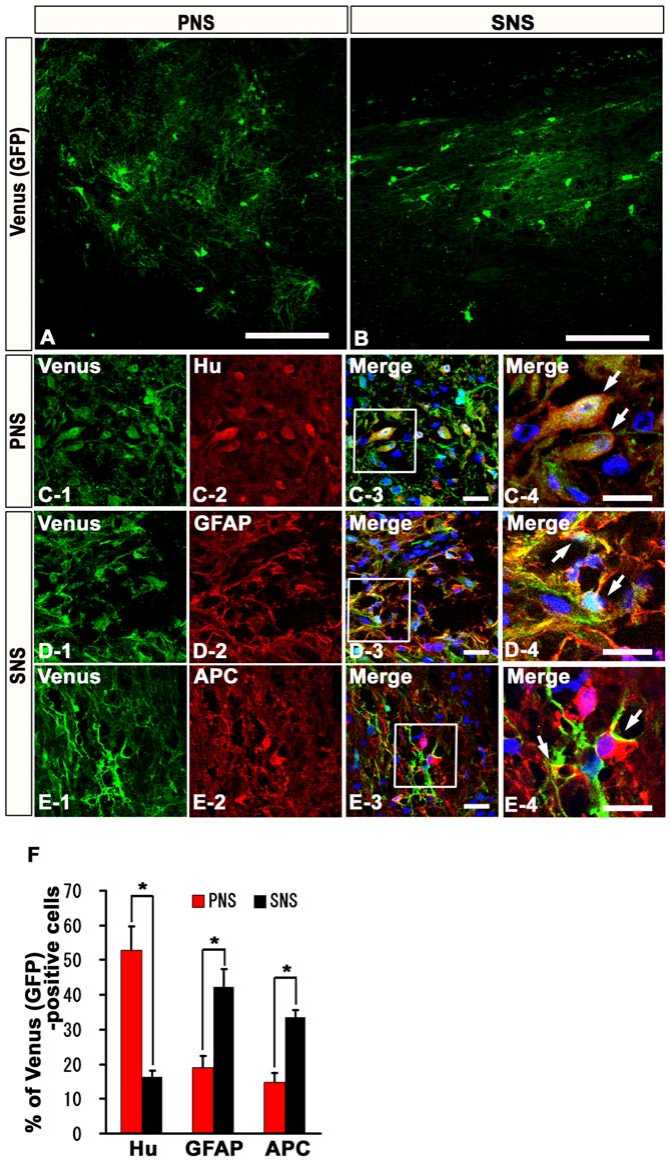
*In vivo* differentiation characteristics of PNS and SNS grafted onto the injured spinal cord. (A)(B) Venus-positive PNS- and SNS-derived grafted cells integrated at or near the lesion epicenter. Venus expression was detected by immunohistochemistry using an antibody against GFP. Scale bar 100 µm. (C)(D)(E) Representative immunohistochemical images of Venus-positive grafted cells that were positive for markers of neural lineages: Hu-positive neurons from PNS group (C), GFAP-positive astrocytes from SNS group (D), and APC-positive oligodendrocytes from SNS group (E). Scale bar: 20 µm. (C-4, D-4, E-4) Higher-magnification image of the boxed areas in C-3, D-3, E-3. Scale bar: 20 µm. (F) The percentage of cell type-specific marker positive cells among the Venus-positive grafted cells, showing the *in vivo* differentiation characteristics of PNS and SNS. The percentage of Hu-positive neurons in the PNS group (52.8±19.1%) was three times that in the SNS group (16.3±5.2%). In contrast, the percentage of GFAP-positive astrocytes or APC-positive oligodendrocytes in the SNS group (42.2±14.4, 33.6±5.4%) was twice that in the PNS group (19.0±9.3, 14.8±7.1%). Values are means ± s.e.m. (*n* = 4). *: *P*<0.05, PNS vs. SNS.

To examine the differentiation characteristics of CCV-PNS and -SNS grafted onto the injured spinal cord, we performed immunohistochemical analyses, and determined the proportion of cells immunopositive for each cell type-specific marker among the Venus-positive grafted cells [3]. Both the PNS- and SNS-derived cells integrated at or near the lesion epicenter and differentiated into Hu-positive neurons, GFAP-positive astrocytes, and APC-positive oligodendrocytes (Fig. 4**A**–**E**). The percentage of Hu/Venus double-positive neurons in the PNS group (52.8±19.1%) was three times that in the SNS group (16.3±5.2%) (Fig. 4**F**). In contrast, the percentage of GFAP/Venus double-positive astrocytes or APC/Venus double-positive oligodendrocytes in the SNS group (42.2±14.4, 33.6±5.4%) was twice that in the PNS group (19.0±9.3, 14.8±7.1%) (Fig. 4**F**) (n = 4). The differentiation patterns of the grafted PNS and SNS were consistent with the results of the *in vitro* differentiation assay, suggesting that PNS and SNS preserved their differentiation tendencies *in vivo*.

### Transplanted SNS, but Not PNS, Enhanced Angiogenesis after SCI

**Figure 5:**
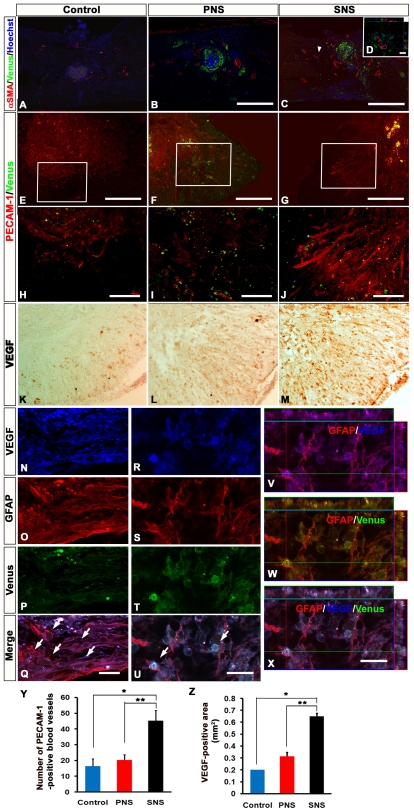
Transplanted SNS, but not PNS, enhanced angiogenesis after SCI. (A)(B)(C) Representative images of αSMA-immunostained sections obtained from the control (A), PNS (B), and SNS (C) groups. Scale bar: 500 µm. (D) Higher-magnification image of the area indicated by the arrowhead in C. Scale bar: 20 µm. While a few αSMA-positive Venus-expressing grafted cells were observed at and near the lesion site in sagittal sections of both the control and PNS groups, significantly more αSMA-positive cells were found in the SNS group, with Venus-positive grafted cells accumulated near the αSMA-positive cells, not colocalized with them. (E)(F)(G) Representative images of PECAM-1-immunostained sections obtained from the Control (E), PNS (F), and SNS (G) groups. Scale bar: 200 µm. (H)(I)(J) Higher-magnification images of the boxed areas in E, F, and G. Scale bar: 100 µm. (K)(L)(M) Representative images of axial sections stained for vascular endothelial growth factor (VEGF). Scale bar: 100 µm. (N–X) Expression of VEGF in GFAP-positive astrocytes among host-derived cells (N–Q) and Venus-positive (GFP immunostained) graft-derived cells (R–X) in the spinal cord from the SNS group (arrows indicate VEGF/GFAP double-positive cells). SNS transplants promoted VEGF expression in both the host- and graft-derived GFAP-positive astrocytes. Scale bar: 20 µm. (Y) Quantitative analysis of blood vessels at the lesion epicenter. PECAM-1 immunostaining revealed similar results, with significantly more PECAM-1-positive blood vessels observed at the lesion site in the SNS group compared with the PNS and control groups. Values are means ± s.e.m. (n = 3).*: P<0.05, Control vs. SNS. **: P<0.05, PNS vs. SNS. (Z) Quantitative analysis of the VEGF-positive area at the lesion epicenter. The VEGF-positive area at the lesion epicenter was significantly broader in the SNS group than in the other groups. Values are means ± s.e.m. (n = 3).*: P<0.05, Control vs. SNS. **: P<0.05, PNS vs. SNS.

To examine the effects of CCV-PNS and -SNS transplantation on angiogenesis after SCI, sagittal and axial sections of the injured spinal cord were examined immunohistochemically for αSMA (a marker for smooth muscle cells) or PECAM-1 (a marker for endothelial cells). While a few αSMA-positive cells were observed at and near the lesion site in sagittal sections of both the control and PNS groups, significantly more αSMA-positive cells were found in the SNS group (Fig. 5**A and B**). These αSMA-positive cells accumulated near Venus-positive grafted cells (Fig. 5**C and D**). Similarly, significantly more PECAM-1-positive blood vessels were observed at the lesion site in the SNS group than in the PNS and control groups (Fig. 5**E**–**J, Y**). To clarify the underlying angiogenic signals, we examined the expression of an angiogenic growth factor, vascular endothelial growth factor (VEGF), in the grafted spinal cord by immunohistochemistry. Although a VEGF-positive area was observed at the lesion epicenter in all three groups 6 weeks after injury (Fig. 5**K**–**M**), it was significantly broader in the SNS group than in the other groups (Fig. 5**Z**). Furthermore, we found many GFAP/VEGF double-positive host astrocytes, which were negative for Venus (GFP) (Fig. 5**N**–**Q**), and a few Venus (GFP)/GFAP-positive graft-derived astrocytes expressing VEGF (Fig. 5**R**–**X**).

### Transplanted SNS, but Not PNS, Promoted Axonal Regrowth and Enhanced Functional Recovery

**Figure 6:**
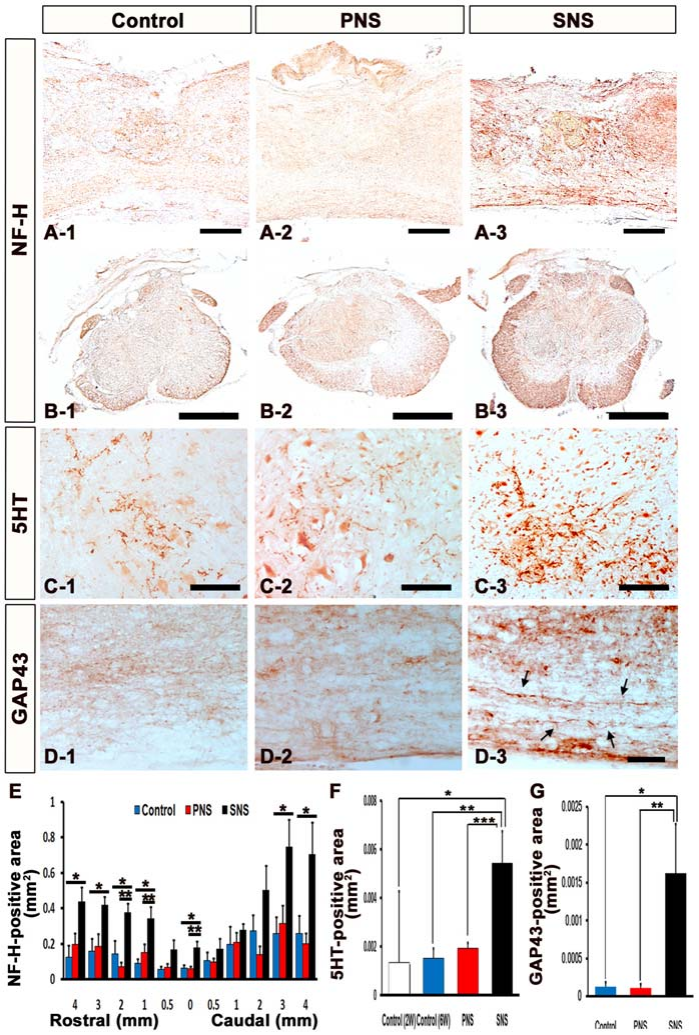
Transplanted SNS, but not PNS, promoted axonal growth. (A) Representative images of sagittal sections stained for NF-H in all three groups. Scale bar: 200 µm. (B) Representative images of axial sections stained for NF-H at the lesion epicenter in all three groups. Scale bar: 500 µm. (C) Representative images of axial sections stained for 5-HT 4 mm caudal to the epicenter from all three groups. Scale bar: 100 µm. (D) Representative images of midsagittal sections stained for GAP43 in the ventral region 1 mm caudal to the epicenter from all three groups, and intact spinal cord. Arrows: GAP43-positive fibers. Scale bar: 50 µm. (E) Quantitative analysis of the NF-H-positive area at each distance point. While few NF-H-positive neuronal fibers were observed at the rim of the lesion epicenter in both the control and PNS groups, there were significantly more NF-H-positive neuronal fibers in the SNS group (B-3) at the lesion epicenter, 1, 2, 3, 4 mm rostral and 3, 4 mm caudal to the lesion epicenter compared with the control group (B-1), and at the lesion epicenter and 1, 2 mm rostral to the lesion site compared with the PNS group (B-2). Values are means ± s.e.m. (*n* = 5). *: *P*<0.05, Control vs. SNS. **: *P*<0.05, PNS vs. SNS. (F) Quantitative analysis of the 5-HT-positive area in axial sections 4 mm caudal to the lesion epicenter. Significantly more 5-HT-positive fibers were observed in the SNS group compared with the other groups. Values are means ± s.e.m. (*n* = 3).*: *P*<0.05, Control (2 weeks after injury) vs. SNS. **: *P*<0.05, Control (6 weeks after injury) vs. SNS. ***: *P*<0.05, PNS vs. SNS. (G) Quantitative analysis of the GAP43-positive area in midsagittal sections in the ventral region 1 mm caudal to the epicenter. Significantly more GAP43-positive fibers were observed in the SNS group than in the other groups. Values are means ± s.e.m. (*n* = 4). *: *P*<0.05, Control vs. SNS. **: *P*<0.05, PNS vs. SNS.

To examine the effects of CCV-PNS and -SNS transplantation on axonal regrowth after SCI, we performed immunohistochemical analyses of the injured spinal cord for NF-H (RT97), 5-hydroxytryptamine (5-HT), and growth-associated protein-43 (GAP43). While few NF-H-positive axons were observed at the rim of the lesion epicenter in the control and PNS groups, there were significantly more NF-H-positive neuronal fibers in the SNS group at the lesion epicenter and perilesional area (Fig. 6**A, B, and E**). 5-HT-positive serotonergic fibers, which are descending raphespinal tract axons [16], [17], were observed at the sites caudal to the lesion epicenter in all three groups 6 weeks after injury (Fig. 6**C**). Quantitative analysis revealed that there were significantly more 5-HT-positive fibers at the site 4 mm caudal to the lesion epicenter (Th10 level), which was approximately at the L1 level, in the SNS group compared with the other groups (Fig. 6F).

While few GAP43-positive axons [18], [19], [20] were detected caudal to the lesion epicenter in the control and PNS groups, there were significantly more GAP43-positive fibers in the SNS group in the ventral region 1 mm caudal to the lesion epicenter (Fig. 6**D, G**), suggesting that transplantation of the gliogenic SNS, but not of the neurogenic PNS, promoted axonal regeneration in the injured spinal cord.

**Figure 7:**
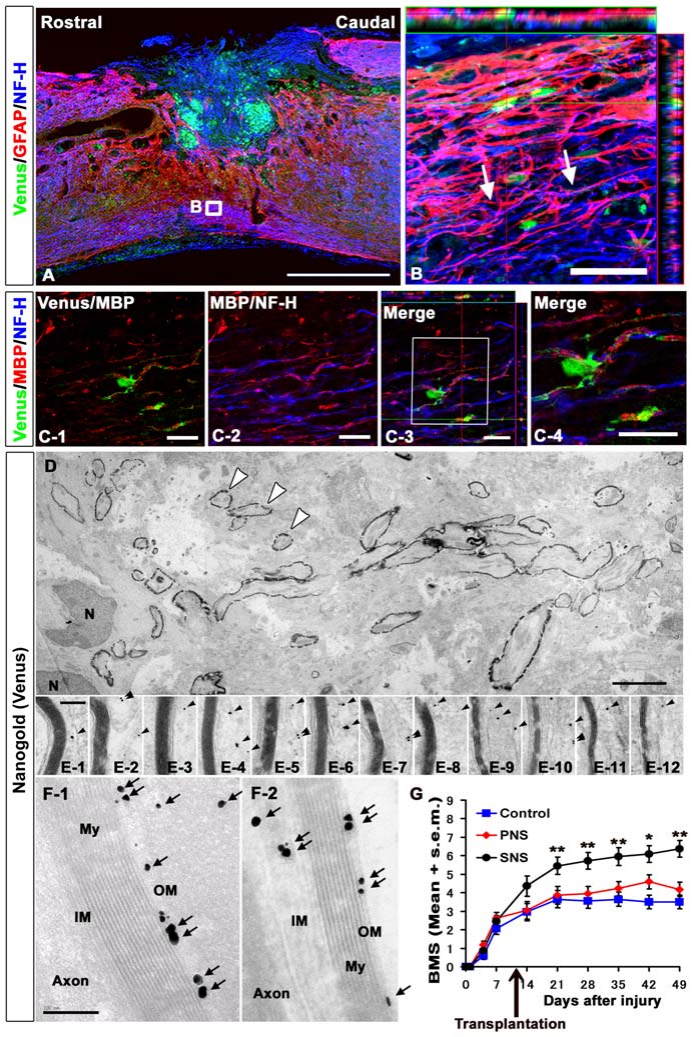
Transplanted SNS, but not PNS, promoted axonal growth, remyelination and functional recovery. (A) NF-H-positive neuronal fibers were observed along with GFAP-positive immature astrocytes derived either from the Venus-positive grafts or the host, and crossed the perilesional area in the SNS group. Scale bar: 50 µm. (B) Higher magnification images of the boxed area in A. Arrows: some examples of axons associated with Venus (GFP)-positive astrocytes. (C) Immunohistochemical analysis of MBP and NF-H in the SNS-transplanted injured spinal cord. SNS-derived Venus-positive cells differentiated into MBP-positive oligodendrocytes, which myelinated NF-H-positive fibers. Scale bar: 20 µm. (D)(E)(F) Representative electron-microscopic images of a remyelination site in sagittal sections from injured spinal cords grafted with SNS, which were immunostained for Venus (grafted cells) using an anti-GFP antibody. Low-magnification images show a group of grafted cells with active remyelination (some indicated by open arrowheads) (D). High-magnification images from (E-1) to (E-12) were obtained from (D), and show various numbers of lamellae in the myelin sheath. The remyelinating transplanted cells were detected as black dots (arrowheads). Venus (GFP)-positive dots (arrows) were localized to the outer and inner mesaxons of the myelin cytoplasm (OM, IM) (F). My: Myelination, N: Nucleus, Scale bar: 5 µm for D, 200 nm for E and F. (G) Mean Basso Mouse Scale for Locomotion (BMS) scores for each group over the 49-day recovery period. Although there was no significant difference in the BMS scores among the control, PNS, and SNS groups on day 14, the SNS group exhibited significantly better functional recovery than the PNS and control groups on day 21 and thereafter. On the other hand, there was no significant difference in the BMS scores between the PNS and control groups. Values are means ± s.e.m. (*n* = 11), *: *P*<0.05, SNS vs. control, 42 days after injury. **: SNS vs. PNS or SNS vs. control on day 21, 28, 35, and 49 after injury.

We also observed NF-H-positive neuronal fibers extending along with the GFAP-positive immature astrocytes, which may have been partially derived from the grafted Venus-positive cells, and crossing the perilesional area in the SNS group (Fig. 7**A and B**). Furthermore, the SNS-derived Venus-positive cells differentiated into MBP-positive oligodendrocytes, which myelinated NF-H-positive fibers (Fig. 7**C**). Electron microscopy also revealed active remyelination in the SNS group (Fig. 7**D**–**F**). The grafted cells were in small groups containing 50–100 cells (Fig. 7**D**). The axons at these sites were enwrapped by myelin sheathes of various thicknesses and numbers of lamellae, which were contributed by the grafted cells, as confirmed by immunolabeling with an anti-GFP antibody to distinguish the transplanted cells from the locally surviving recipient cells (Fig. 7**E**). A much higher magnification revealed nanogold-labeled Venus-positive spots in the outer and inner mesaxons of the myelin cytoplasm. Some axons close to the lesion epicenter had undergone considerable re-myelination, and were enwrapped in spirals of more than ten layers of compacted lamellae (Fig. 7**F**).

Finally, we monitored the locomotor functional recovery in all three groups using the BMS scoring scale [21]. The contusive SCI resulted in complete paralysis (BMS score 0) on day 1, followed by gradual recovery with a plateau around 3 weeks. Although there was no significant difference in the BMS scores among the control, PNS, and SNS groups on day 14, the SNS group exhibited significantly better functional recovery than the PNS and control groups on day 21 and thereafter. On the other hand, there was no significant difference in the BMS scores between the PNS and control groups (Fig. 7**G**). From a clinical perspective, the recovery of the SNS group to levels exhibiting frequent to consistent weight-supported plantar steps and occasional forelimb-hindlimb coordination was especially noteworthy.

## Discussion

Methods for effectively inducing neural differentiation from pluripotent ES cells have been extensively studied [6] and are expected to be applied in cell replacement therapies for SCI [22]. However, detailed investigations of the optimal cell sources for promoting recovery from SCI are lacking. We recently developed an ES cell culture system that recapitulates the temporal progression of NS/PCs from the FGF-responsive early neurogenic NS/PCs to the EGF-responsive late gliogenic NS/PCs, consistent with CNS development *in vivo* [10], [23] (Fig. 1**G, H**). Taking advantage of this difference in differentiation tendency, here we examined the distinct effects of the neurogenic PNS and gliogenic SNS on recovery following SCI.

One of the mechanisms underlying this developmental stage-dependent gliogenic transition of NS/PCs is the epigenetic regulation of glial cell-specific genes. The gradual demethylation of CpGs around the Stat3 recognition sequence in the GFAP promoter is thought to be involved in the developmental stage-dependent increase in transcription of the GFAP gene and the acquisition of astrocytic differentiation potentials [24], [25], [26]. Interestingly, this process is also observed in our ES cell-derived neurosphere system, in which the proportion of unmethylated CpGs in this region gradually increases during the development of ES cells into secondary neurospheres [10]. This may explain why the *in vitro* differentiation potentials of both the PNS and SNS were preserved even after their transplantation into injured spinal cord, despite its gliogenic environment (Fig. 4**A**–**F**) [27]. Since there was no significant difference in the numbers of grafted PNS and SNS in the injured spinal cord 6 weeks after transplantation, the difference in the in vivo differentiation potentials of the grafted neurospheres was the critical factor influencing the functional recovery after SCI. More than 70% of the grafted SNS cells differentiated into GFAP-positive astrocytes or APC-positive oligodendrocytes, and the engraftment of these cells led to improved functional recovery (Fig. 4**F**). In contrast, engrafted PNS cells, which mainly differentiated into neurons, did not promote functional recovery.

Determining the exact mechanisms through which the transplanted SNS, or glial cells, improved the recovery of the traumatically injured CNS has been challenging. The engrafted SNS cells could promote a wide range of effects, and here we showed positive effects of their transplantation on tissue sparing, myelination, angiogenesis, and axonal regeneration compared with the control group, and on myelination, angiogenesis, and axonal regeneration compared with the PNS group. One possible explanation for the functional recovery observed in the SNS group is that the SNS-derived astrocytes provided axonal guidance cues. This idea is supported by previous studies in which glial progenitors or glial progenitor-derived astrocytes were engrafted [28], [29], [30], [31]. Immature astrocytes purified from the postnatal CNS have been shown to promote extensive neurite growth from a variety of neurons [32], [33].

Although the reactive astrocytes in glial scar tissue express proteoglycans that can inhibit axonal growth, and have been shown to play a major role in the formation of misaligned scar tissue at sites of injury [34], [35], [36], [37], [38], we and others have previously shown that reactive astrocytes also have pivotal roles in the repair of injured tissue and recovery of motor function in the subacute phase after SCI, by sealing off injured areas and preventing the further spread of damage. They also produce an array of neurotrophic and growth factors [39]. Moreover, some astrocytes in the host spinal cord acquire stem-cell properties after injury and hence represent a promising cell type for initiating repair [40]. In combination with host astrocytes, immature astrocytes generated by the grafted SNS may express axonal growth-supporting molecules such as laminin, fibronectin, nerve growth factor (NGF), neurotrophin-3 (NT-3), vasoactive intestinal polypeptide (VIP), and activity-dependent neurotrophic factor (ADNF) [41] with minimal expression of chondroitin sulfate proteoglycans (CSPGs) [42]. In addition, SNS transplantation 9 days after SCI, between the acute and chronic phases, is likely to prevent grafted cells from differentiating into glial scar-forming reactive astrocytes due to their minimal expression of cytokines [3], [43] and instead generate immature astrocytes, which provide cues for axonal regeneration. In fact, our immunohistochemical analysis revealed NF-H-positive neuronal fibers aligned with GFAP-positive fibers within the lesion site of the SNS group, suggesting that the SNS transplants promoted the alignment of regenerating axons with the fibers of astrocytes, which in turn promoted axonal growth into and out of the SNS grafts (Fig. 7**A and B**). In addition, the 5-HT-raphespinal system of the spinal cord has been shown to represent axonal regeneration after spinal cord injury [16], [17], and the apparent regeneration and/or sparing of host 5-HT-positive fibers elicited by the grafting of SNS may have contributed to the observed functional recovery, since these fibers were not observed in the control or PNS groups (Fig. 6**C and F**).

Another possible explanation for the functional improvement in the SNS group is the enhancement of angiogenesis, since angiogenesis is reported to promote endogenous repair and support axonal outgrowth after SCI [44]. Under hypoxic conditions, astrocytes express angiogenic growth factors, including VEGF [45], [46]. We revealed that transplanted SNS, but not PNS, enhanced angiogenesis after SCI (Fig. 5**A–Z**). We observed many host astrocytes (Fig. 5**N–** **Q**), and a few SNS-graft-derived astrocytes that expressed VEGF (Fig. 5**R–** **X**), suggesting that the SNS transplants promoted VEGF expression in both the host- and graft-derived GFAP-positive astrocytes. The increase in blood vessels elicited by the transplantation of ES cell-derived gliogenic NS/PCs may have improved axonal growth and prevented atrophy of the injured spinal cord.

The functional improvement might also be due to remyelination by SNS-derived oligodendrocytes, as supported by previous transplantation studies of ES cell-derived NS/PCs or oligodendrocyte progenitor cells (OPCs) [8], [47]. While the neurogenic PNS dominantly differentiated into Hu-positive neurons (Fig. 4**F**), the gliogenic SNS differentiated into APC-positive oligodendrocytes that provided MBP-positive sheathes and promoted myelination after SCI (Fig. 2**C, D, F**, and Fig.7**C–** **F**).

In summary, here we took advantage of our recently established neurosphere-based culture system of ES cell-derived NS/PCs, in which PNS and SNS exhibit neurogenic and gliogenic potentials, respectively, and found that SNS cells were the most effective for promoting recovery after SCI. We showed that grafted SNS generated approximately equal numbers of GFAP-positive astrocytes and APC-positive oligodendrocytes in vivo. Both of these glial cell types may have contributed to the functional recovery, through trophic effects and the promotion of angiogenesis and axonal regeneration by immature astrocytes, and possibly through remyelination by grafted oligodendrocyte progenitor cells. Notably, the transplantation of PNS did not improve the functional recovery after SCI. These findings provide critical information for clinical trials using human ES- and induced pluripotent stem cell (iPS)-derived NS/PC transplantation for SCI.

Moreover, our results suggest that ES cell-derived NS/PCs cultured for relatively long periods may provide sufficient amounts of efficient glial donor cells for cell transplantation therapies. This strategy may also prevent the contamination of tumorigenic undifferentiated ES cells that occurs during long-term culture under serum-free conditions, and support the development of safe embryonic stem cell-based treatment strategies for spinal cord injury. Although both the CCV-PNS- and CCV-SNS-derived Venus-positive cells survived without forming tumors for 6 weeks after transplantation in this study, careful observation for a longer period will be necessary to assess the possibility of tumor formation.

In the near future, other types of pluripotent stem cells, such as nuclear transfer ES (ntES) and iPS cells, which avoid the risk of immunological rejection and ethical concerns, will need to be evaluated to examine the applicability of human ES cells and human iPS cells in clinical applications.

## Materials and Methods

### ES Cell Culture and Differentiation

Mouse ES cells (EB3) [13] grown on gelatin-coated (0.1%) tissue-culture dishes were maintained in standard ES cell medium and used for EB formation as previously described, with slight modifications [9], [13], [48]. For neural induction, ES cells were dissociated into single cells with 0.25% trypsin-EDTA and cultured in bacteriological dishes for 6 days, to allow the formation of EBs. A low concentration of RA (low-RA; 10^−8^ M, Sigma) was added on day 2 of EB formation. The EBs were dissociated into single cells with 0.25% trypsin-EDTA and cultured in suspension at 5×10^4^ cells/ml for 7 days in Media hormone mix (MHM) medium with 20 ng/ml FGF2 (Peprotech) and 2% B27 (Invitrogen), to obtain primary neurospheres (PNS). These PNS were dissociated into single cells with TripleLE Select (Invitrogen) and cultured again in suspension at 5×10^4^ cells/ml for 7 days under the same conditions, to form secondary neurospheres (SNS) (Fig. 1**B**) [10]. For differentiation analysis, PNS and SNS were allowed to differentiate on poly-L-ornithine/fibronectin-coated coverslips for 5 days, followed by immunocytochemistry. The frequency of colonies consisting of βIII tubulin-positive neurons, GFAP-positive astrocytes, and O4-positive oligodendrocytes (N, A, O: colonies containing more than 20 positive cells are in capital letters; n, a, o: colonies containing fewer than 19 cells are in lower-case letters) is presented as the percentage of total colonies (50 colonies each) from three independent experiments.

### Transfection of CAG-CBRluc-IRES-Venus

To visualize transplanted cells by both fluorescence and luminescence, we established an ES cell line that constitutively expresses a click beetle red-emitting luciferase variant (CBR*luc*) [11] and Venus [a yellow fluorescence protein (YFP) mutant] [12] by transfecting a linearized CAG-CBR*luc*-IRES-Venus plasmid (CCV; Fig. 1**A**) into EB3 ES cells using lipofectamine2000 (Invitrogen). Stably transfected ES cells were selected by G418 (200 µg/ml), subcloned, and screened by the expression of both CBR*luc* and Venus. The Venus could be detected by antibodies against EGFP.

### Flow Cytometry

Undifferentiated ES cells, PNS, and SNS were dissociated and processed for flow cytometric analysis by FACS Calibur (Becton-Dickinson). The Venus-positive cells were counted and are presented as the percentage of the total number of cells, excluding dead cells stained by propidium iodide.

### Spinal Cord Injury Model and Transplantation

Adult female C57BL/6J mice (20–22 g) were anesthetized via intraperitoneal (i.p.) injection of ketamine (100 mg/kg) and xylazine (10 mg/kg). After laminectomy at the 10th thoracic spinal vertebra (T10), a contusive SCI was induced at the same level using a commercially available SCI device (IH impactor, Precision Systems and Instrumentation, Lexington, KY), as described previously [49]. This device creates a reliable contusion injury by rapidly applying a force-defined impact (60 kdyn) with a stainless steel-tipped impounder. The initial touch point of the impactor with the dura was determined (using the vibrator mode of the impactor tip), and from there a 1.5-mm displacement was applied to the spinal cord. Force curve readings revealed an average value of 63±0.5 kdyn.

Nine days after the injury, CCV-PNS (n = 11) or -SNS (n = 11) that had been cultured for 7 days were partially dissociated and transplanted into the lesion epicenter using a glass micropipette (5×10^5^ cells/mouse) and stereotaxic injector (KDS 310, Muromachi-kikai, Tokyo, Japan). An equal volume of PBS was injected into the control group (n = 11). Hind limb motor function was evaluated for 6 weeks after SCI using the locomotor rating test of the Basso-Mouse-Scale (BMS), as described previously [21]. Well-trained investigators, blinded to the treatments, performed the behavioral analysis, determining the BMS scores at the same time each day. All animal experiments were approved by the ethics committee of Keio University, and were in accordance with the Guide for the Care and Use of Laboratory Animals (National Institutes of Health, Bethesda, MD).

### Bioluminescence Imaging (BLI)

BLI was performed using a Xenogen-IVIS 100 cooled CCD optical macroscopic imaging system (SC BioScience, Tokyo, Japan) [3], [50]. To examine the effective expression of CBR*luc in vitro*, we used a CCD-based macroscope detector to determine the luminescence intensity of cultures with various numbers of cells (0 to 5×10^5^ cells per well) in the presence of D-luciferin (150 µg/ml). The integration time was fixed at a 5-min duration for each image, and the signals were reported as photons/cells/sec. For *in vivo* BLI, D-luciferin was injected i.p. into mice (150 mg/kg body weight), and serial images were acquired 15–40 min. later, until a maximum signal intensity was obtained with the field-of-view, which was set at 15 cm. We found this time window to be optimal, since the signal intensity peaked 15–40 min after D-luciferin administration, and was followed by a 15-min plateau (data not shown). All images were analyzed with Igor (WaveMetrics, Lake Oswego, OR) and Living Image software (Xenogen, Alameda, CA), and the optical signal intensity was expressed as photon flux, in units of photons/sec/cm^2^/steradian. The results were displayed as a pseudocolor photon count image superimposed on a grayscale anatomic image. To quantify the measured light, we defined a region of interest (ROI) over the cell-implanted area and examined all the values within the same ROI. The signal intensity of the engrafted cells was measured weekly for 6 weeks after transplantation.

### Histological Analyses

Animals were anesthetized and transcardially perfused with 4% paraformaldehyde in 0.1 M PBS 6 weeks after transplantation. The spinal cords were removed, embedded in OCT compound (Sakura Finetechnical Co., Ltd.), and sectioned in the sagittal/axial plane at 20 µm on a cryostat (Leica CM3050 S). The injured spinal cords from the three groups were histologically evaluated by Hematoxylin-eosin (H-E) staining, Luxol Fast Blue (LFB) staining, and immunohistochemistry. The injured spinal cord from the vehicle control group 2 weeks after SCI was also evaluated by LFB staining and immunohistochemistry for 5-HT. Both cultured cells and tissue sections were stained with the following primary antibodies: anti-GFP (rabbit IgG, 1∶500, MBL), anti-βIII tubulin (mouse IgG, 1∶1000, Sigma), Alexa488-conjugated anti-βIII tubulin (mouse IgG, 1∶4000, Covance), anti-Hu (human IgG, 1∶1000, a gift from Dr. Robert Darnell, The Rockefeller University), anti-GFAP (rabbit IgG, 1∶4000, Dako), anti-GFAP (guinea pig IgG, 1∶4000, Advanced Immunochemical Inc.), anti-GFAP (rat IgG, 1∶200, Invitrogen), anti-O4 (mouse IgM, 1∶5000, Chemicon), anti-APC CC-1 (mouse IgG, 1∶100, Calbiochem), anti-MBP (chicken IgY, 1∶200, Aves Labs), anti-Neurofilament RT97 (NF-H, mouse IgG, 1∶200, Chemicon), anti-5-HT (goat IgG, 1∶200, Immunostar), anti-GAP43 (mouse IgG, 1∶2000, Chemicon), Cy3-conjugated anti-SMA (mouse IgG 1∶500, Sigma), anti-PECAM-1 (rat IgG, 1∶50, BD Bioscience Pharmingen), and anti-VEGF (rabbit IgG, 1∶50, Santa Cruz Biotechnology).

For immunohistochemistry with anti-Venus, VEGF, -NF-H, -5-HT, and -GAP43 antibodies, we used a biotinylated secondary antibody (Jackson Immunoresearch Laboratory, Inc.), after exposure to 0.3% H_2_O_2_ for 30 minutes at room temperature to inactivate endogenous peroxidase. The signals were enhanced with the Vectastain ABC kit (Vector Laboratories, Inc.). Nuclei were stained with Hoechst33258 (10 µg/ml, Sigma). The samples were examined with a universal fluorescence microscope (Axiocam, Carl Zeiss) or a confocal laser scanning microscope (LSM510, Carl Zeiss).

For immunoelectron microscopy, frozen sections were incubated with nanogold-conjugated anti-rabbit secondary antibody (1∶100 Invitrogen) followed by incubation with the primary anti-GFP antibody. After enhancement with HQ-Silver kit (Nanoprobes Inc.), sections were postfixed with 0.5% osmium tetroxide, dehydrated through ethanol, and embedded in Epon. Ultrathin sections were stained with uranyl acetate and lead citrate, observed under a transmission EM (JEOL model 1230), and photographed with a Digital Micrograph 3.3 (Gatan Inc.).

### Quantitative Analyses of Stained Tissue Sections through Transplanted Spinal Cord

To quantify HE-, LFB-, or immunostained sections, images were obtained by a universal fluorescence microscope (Axiocam, Carl Zeiss), manually outlined, and quantified by Micro Computer Imaging Device (MCID; Imaging Research Inc., St. Catharines, Ontario, Canada). Constant threshold values were maintained for all the analyses with MCID. HE-stained images were taken at the lesion epicenter and 2, 1, and 0.5 mm rostral and caudal to the epicenter in axial sections at ×25 magnification (n = 5, each). To analyze the LFB-positive area after transplantation, we automatically captured four regions from each animal in axial sections at the lesion epicenter and 2 mm and 1 mm rostral and caudal to the epicenter at ×200 magnification. Analyses were performed 2 weeks or 6 weeks after SCI for the vehicle-control group and 6 weeks after for the PNS and SNS groups. The total myelinated area was quantified by MCID using light intensity gain counting (n = 3, each). For the Venus (GFP)-positive area after transplantation, we captured in midsagittal sections the epicenter at ×25 magnification from each animal (6 weeks after SCI for the vehicle-control, PNS, and SNS groups), and quantified the total Venus-positive area (n = 6, each). NF-H-stained images were taken at the epicenter and 4, 3, 2, 1, and 0.5 mm rostral and caudal to the epicenter in axial sections at ×50 magnification, and the NF-H-positive areas were quantified using light intensity gain counting (n = 5, each). VEGF-stained images were taken at the lesion epicenter in axial sections at ×50, and the VEGF-positive areas were quantified using light intensity gain counting (n = 3, each). To analyze the 5-HT-positive area after transplantation, we automatically captured five regions from each animal in axial sections 4-mm caudal to the lesion epicenter (Th10 level), which was approximately at the L1 level, a non-lesion site, at ×200 magnification. The analysis was performed 2 weeks or 6 weeks after SCI for the vehicle-control group and 6 weeks after for the PNS and SNS groups. The total 5-HT-positive area was quantified (n = 3, for each condition). For the GAP43-positive area after transplantation, we captured the ventral regions in midsagittal sections 1 mm caudal to the epicenter at ×200 magnification from each animal (6 weeks after SCI for the vehicle-control, PNS, and SNS groups), and quantified the total GAP43-positive area (n = 4). To quantify the proportion of cells positive for each cell type-specific marker *in vivo*, we selected representative midsagittal sections and automatically captured five regions within 500 µm rostral and caudal to the lesion epicenter at ×200. The engrafted cells in each section that were positive for both Venus and each cell type-specific marker were counted (n = 4). The PECAM-1-positive blood vessels were counted manually in axial sections of the lesion epicenter at ×200 magnification (n = 3, each).

### Statistical Analysis

All data are presented as the mean ± s.e.m. An unpaired two-tailed Student's *t*-test was used for the BLI analyses and Venus-stained analysis, and *in vivo* differentiation assays. ANOVA followed by the Turkey-Kramer test for multiple comparisons among the three transplantation groups was used for the *in vivo* differentiation analysis and PECAM-1-, VEGF-, 5HT-, and GAP43-stained analysis. Repeated measures two-way ANOVA followed by the Turkey-Kramer test was used for HE-, LFB-, NF-H-stained and BMS analysis. Pearson's correlation coefficient was used for correlation of the results of BLI analysis and the quantification of Venus-positive area. In all statistical analyses, the significance was set at *P*<0.05.
